# A network meta-analysis of therapeutic and prophylactic management of vasospasm on aneurysmal subarachnoid hemorrhage outcomes

**DOI:** 10.3389/fneur.2023.1217719

**Published:** 2023-08-17

**Authors:** Benjamin Chousterman, Brice Leclère, Louis Morisson, Yannick Eude, Etienne Gayat, Alexandre Mebazaa, Raphael Cinotti

**Affiliations:** ^1^Department of Anesthesia and Critical Care, Hôpital Lariboisière, Assistance Publique des Hôpitaux de Paris, University Hospital of Saint-Louis-Lariboisière, Paris, France; ^2^UMR 942 MASCOT, Hôpital Lariboisière, Assistance Publique des Hôpitaux de Paris, Paris, France; ^3^Public Health Department, Hôpital Saint-Jacques, University Hospital of Nantes, Nantes, France; ^4^MiHAR, IRS 2, University of Nantes, Nantes, France; ^5^Department of Anesthesia and Pain Medicine, Hôpital Maisonneuve-Rosemont, CIUSSS de l'Est de l'Ile de Montréal, Boulevard de l'Assomption, University of Montréal, Montréal, QC, Canada; ^6^Department of Anesthesia and Critical Care, Hôtel-Dieu, University Hospital of Nantes, Nantes, France; ^7^UMR 1246 SPHERE MethodS in Patients-Centered Outcomes and Health Research, Institut de Recherche en Santé 2, Nantes, France

**Keywords:** subarachnoid hemorrhage, network meta-analysis, mortality, outcome, vasospasm, delayed ischemic deficit

## Abstract

**Background:**

Vasospasm and cerebral ischemia after aneurysmal subarachnoid hemorrhage are associated with mortality and poor neurological outcomes. We studied the efficacy of all available strategies targeting vasospasm and cerebral ischemia on outcomes in a network meta-analysis.

**Methods:**

We searched EMBASE and MEDLINE databases from 1 January 1990 and 28 November 2021 according to PRISMA guidelines. Randomized controlled trials and longitudinal studies were included. All curative or preventive strategies targeting vasospasm and/or cerebral ischemia were eligible. A network meta-analysis was performed to compare all interventions with one another in a primary (randomized controlled trials only) and a secondary analysis (both trials and longitudinal studies). Mortality by 3 months was the primary outcome. Secondary outcomes were vasospasm, neurological outcome by 3 months, and dichotomized as “good” or “poor” recovery according to each study definition.

**Results:**

A total of 2,382 studies were screened which resulted in the selection of 192 clinical trials (92 (47.9%) and 100 cohorts (52.1%) and the inclusion of 41,299 patients. In randomized controlled studies, no strategy decreased mortality by 3 months. Statins (0.79 [0.62–1]), tirilazad (0.82 [0.69–0.97]), CSF drainage (0.47 [0.29–0.77]), and clazosentan (0.51 [0.36–0.71]) significantly decreased the incidence of vasospasm. Cilostazol was the only treatment associated with improved neurological outcomes by 3 months in the primary (OR 1.16, 95% CI [1.05–1.28]) and secondary analyses (OR 2.97, 95% CI [1.39–6.32]).

**Discussion:**

In the modern era of subarachnoid hemorrhage, all strategies targeting vasospasm failed to decrease mortality. Cilostazol should be confirmed as a treatment to improve neurological outcomes. The link between vasospasm and neurological outcome appears questionable.

**Systematic review registration:**

https://www.crd.york.ac.uk/PROSPERO/display_record.php?RecordID=116073, identifier: PROSPERO CRD42018116073.

## 1. Introduction

Aneurysmal subarachnoid hemorrhage (SAH) bears substantial mortality and morbidity (1), with an estimated cost of €38,000 per patient in the 1st year of illness in 2010 (2). Vasospasm and/or delayed cerebral ischemia have been identified for decades as early complications and thus potential therapeutic targets (3). Nimodipine demonstrated efficacy in decreasing neurological impairment after SAH by potentially decreasing vasospasm-related cerebral ischemia (4). Since then, numerous preventive and curative treatments targeting vasospasm or delayed cerebral ischemia have been tested. These strategies mainly focus on 1) improving cerebral blood flow [triple-H therapy (5)], 2) improving intracerebral macrovascular remodeling [magnesium (6), statins (7), and nimodipine (8)], 3) decreasing the occurrence of vasospasm with intracranial blood removal (9), and finally, 4) modulating the neuro-inflammatory response and ischemia ptirilazad (10) and corticosteroids (11)]. The results have been conflicting since a decrease in vasospasm (12) does not always lead to neurological improvement (13). There have, therefore, been few significant bedside management modifications on vasospasm/cerebral ischemia, and no specific treatment was recommended in the latest guidelines (14).

We hypothesized that preventive and curative strategies targeting vasospasm and/or cerebral ischemia do not equally alter the outcome after SAH. We performed a systematic review to explore the effect on mortality by day 90 of all interventions that have been tested in the prevention and treatment of vasospasm and/or cerebral ischemia after SAH in a network meta-analysis enabling comparison of the efficacy of the various treatments. Vasospasm, delayed cerebral ischemia, neurological outcome by 3 months, mortality, and outcomes beyond 3 months were secondary outcomes.

## 2. Methods

### 2.1. Literature research and study selection

This is a systematic review and a network meta-analysis of prevention and curative treatments of vasospasm/brain ischemia in SAH patients. We searched the EMBASE and MEDLINE databases for articles written in English or in French that contained the following terms: “subarachnoid hemorrhage,” “treatment outcome,” “outcome assessment,” and “mortality.” The extensive search string added more terms (e.g., “disease management”) and is available in Supplementary Text S1. Since the diagnosis of vasospasm or brain ischemia can be challenging and not always specifically targeted (15), very broad research and selection of studies were undertaken in order to evaluate all strategies implemented in the acute phase of SAH without specifically targeting these complications (7). The meta-analysis was performed according to PRISMA guidelines.

We included trials and observational studies that were published between 1 January 1990 and 28 November 2021. In addition, these studies focus on adult patients (>18 years) with documented ruptured SAH, undergoing a therapeutic strategy in order to prevent or treat vasospasm and/or delayed ischemic deficits. Studies focusing on securing the aneurysm (clip or coil) were not included in the review (PROSPERO CRD42018116073).

The reference lists of all selected articles and previous meta-analyses were checked for additional references (snowballing). Related articles that cited the selected studies were searched in Google Scholar^®^ (reverse snowballing). Surgical, radiological, anesthetic, medical, and critical care management were performed according to institutional protocols. Three authors of this study (BC, RC, and LM) checked all titles and abstracts identified from the search and assessed all randomized clinical trials and observational studies for eligibility. After the selection of potential studies, independent verification was performed separately by two authors. In case of disagreement, the third author checked for eligibility. Persistent disagreement was resolved by discussion.

### 2.2. Data extraction and quality assessment

One author (RC) designed a standard extraction form, and four others (BC, LM, BL, and YE) amended and validated the design of the form before data extraction. Three authors (BC, RC, and LM) separately extracted the following data from each available study: first author, title, year of publication, study design, quality of the study (Jaddad score for trials and Newcastle–Ottawa score for longitudinal observational studies), therapeutic strategy (see below), neurologic baseline severity (World Federation of Neurosurgeon, Hunt and Hess, Fisher scores), use of surgical clip or coil, prophylactic or curative treatment, site of administration (enteral, intravenous, and intrathecal), incidence and definition of vasospasm (clinical, Doppler, angiography, and CT scan), incidence of delayed cerebral ischemia, hospital length of stay, in-hospital mortality, withdrawal of life sustaining therapies, mortality at 3, 6, and 12 months, good neurological recovery at 3, 6, and 12 months, and details of neurological scales whenever available (modified Rankin Scale, Glasgow Outcome Scale, and Glasgow Outcome Scale-Extended) on hospital discharge, 3 months, 6 months, or 12 months. We contacted the authors when data were incomplete or unavailable.

Initially, the following therapeutic strategies were observed in the included studies: magnesium, statin, triple H therapy, volume status management, cardiac output management, anti-endothelin, neurosurgical management, neuroradiological intervention, intrathecal fibrinolysis, nimodipine, other calcic inhibitors, beta-blockers, cilostazol, fasudil, transfusion, erythropoietin, corticosteroids, antioxidant, tirilazad, hypothermia, immunomodulation therapies, and sodium management. To enable the network meta-analysis, some strategies were merged: triple H therapy, vascular expansion strategies, and cardiac output management were pooled in the “cardiovascular management” group, and transfusion and erythropoietin were also merged together. Miscellaneous anti-inflammatory treatments (antioxidant and interleukin) were pooled into the “inflammation” group. Data were extracted from the tables, figures, and text of the manuscripts of the selected articles, and collected using an online Sphynx^®^ CRF.

### 2.3. Outcomes

The primary outcome was mortality by 3 months. Secondary outcomes were the incidence of vasospasm, the incidence of delayed cerebral ischemia, mortality by 6 months and later, neurological outcomes at various time points (3 months, 6 months, and later). In each study, the definition of a good or poor outcome could be different according to the scale that the authors used (modified Rankin Scale, Glasgow Outcome Scale, or Glasgow Outcome Scale-Extended mostly). We retained the authors' definitions of good or poor neurological outcomes.

### 2.4. Statistical analysis

Analyses were performed on data reported in the individual studies. No imputations were made regarding unreported data. When available, we analyzed the intention-to-treat population. Since we wanted to compare multiple therapeutic strategies, we relied on network meta-analyses. Several different network meta-analyses were performed, one for each primary and secondary outcome, using the same overall approach. First, we made sure that all of the evaluated therapeutic strategies belonged to a unique network. The remaining studies were used to estimate pooled odds ratios (ORs) for each strategy against placebo using the inverse variance method. This method enabled us to compute the I^2^ statistics, which represents the percentage of variation across studies that is due to heterogeneity rather than chance. A high level of heterogeneity is a sign that the included studies are too different to be used to estimate a coherent pooled estimation. Both fixed and random effects models are estimated by the inverse variance method. Given the low level of heterogeneity that we observed for vasospasm in randomized controlled trials and mortality and neurological outcome, we chose to report only the results of the fixed effect models.

As planned (CRD42018116073), the primary network **outcome** was performed on mortality by 3 months. Secondary **outcomes** were neurological outcomes by 3 months, vasospasm, delayed brain ischemia, and mortality and neurological outcome ≥3 months. In the primary analysis, we included only randomized controlled trials since observational studies do not provide the same level of scientific evidence. We performed a secondary analysis by rerunning the analyses on all selected trials (controlled trials, cohorts, or observational studies) for mortality and neurological outcomes by 3 months. No analysis was performed regarding mortality and neurological outcomes after 6 months, owing to the vast heterogeneity and low reporting of this outcome throughout the studies. The presence of a potential publication bias was assessed using a funnel plot and an asymmetry test based on rank correlation (16). All statistical analyses were performed in R version 4.2.0. The network meta-analysis relied on the *netmeta* package [Gerta Rücker, Ulrike Krahn, Jochem König, Orestis Efthimiou, and Guido Schwarzer (2021); *netmeta*: Network Meta-Analysis using Frequentist Methods. R package version 2.6-0. https://CRAN.R-project.org/package=netmeta] (16). This article was written according to PRISMA guidelines (Preferred Reported Items for Systemic Reviews and Meta-Analysis).

### 2.5. Standard protocol approvals and patient consent

All the included studies were approved by the appropriate ethics committees.

### 2.6. Data availability statement

Data not provided in the article may be shared (anonymized) at the request of any qualified investigator for purposes of replicating procedures and results.

## 3. Results

### 3.1. Data selection and quality

**Figure 1:**
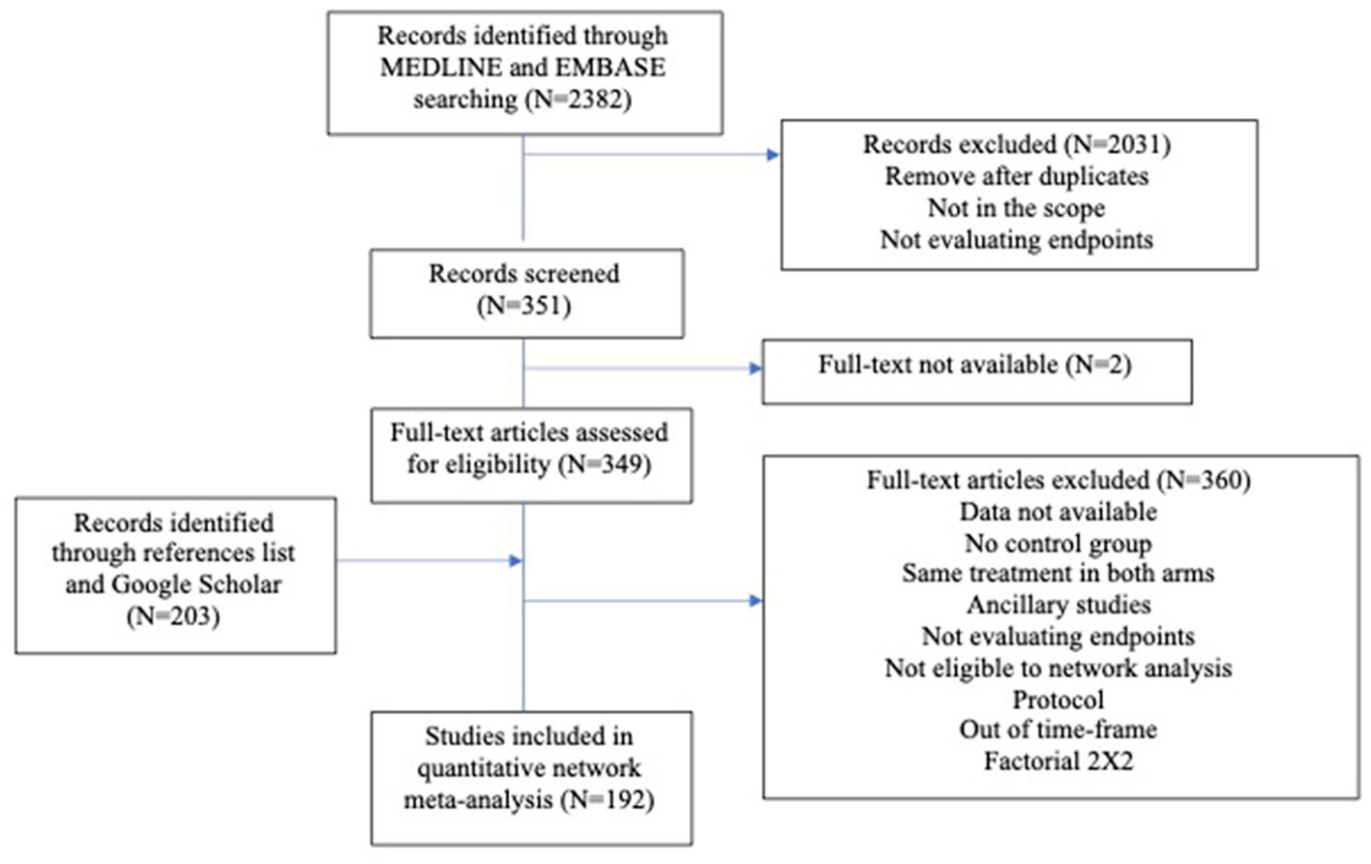
Flowchart of the study.

Out of 2,382 references, 192 studies involving 41,299 patients met the inclusion criteria and were included in the literature review. The selection process and the main therapeutic strategies are detailed in Figure 1. In total, 92 studies (47.9%) were clinical trials, and 100 (52.1%) were observational studies. Randomized controlled studies included 18,951 patients, and observational studies included 22,348 patients. Among clinical trials, 40.2% had a Jaddad score of 4–5. Among observational studies, 20 had a Newcastle–Ottawa score of 0–2 stars, 16 had a score of 3–5 stars, and 60 had a score of 6–9 stars. The median score was 6 [4–8]. The complete list of studies included in the meta-analysis is available in Supplementary Table S2 (List of included studies). The number of studies included per decade is available in Supplementary Figure S3.

### 3.2. Study populations

Among interventional studies, the population size varied from 12 to 1,203 with a median of 93. The size of observational studies varied from 15 to 6,418 with a median of 104. In the overall population, 11,999 (29%) patients underwent aneurysm clipping, and 4,183 **(10.1%)** underwent aneurysm coiling. The overall mortality rate was equal to 11% by 1 month and 14% by 3 months. The overall proportion of good neurological outcomes was equal to 57.2% by 1 month and 57.9% by 3 months. The therapeutic strategy was prophylactic in 90 (46.9%) studies.

### 3.3. Primary outcome: mortality by 3 months

**Figure 2:**
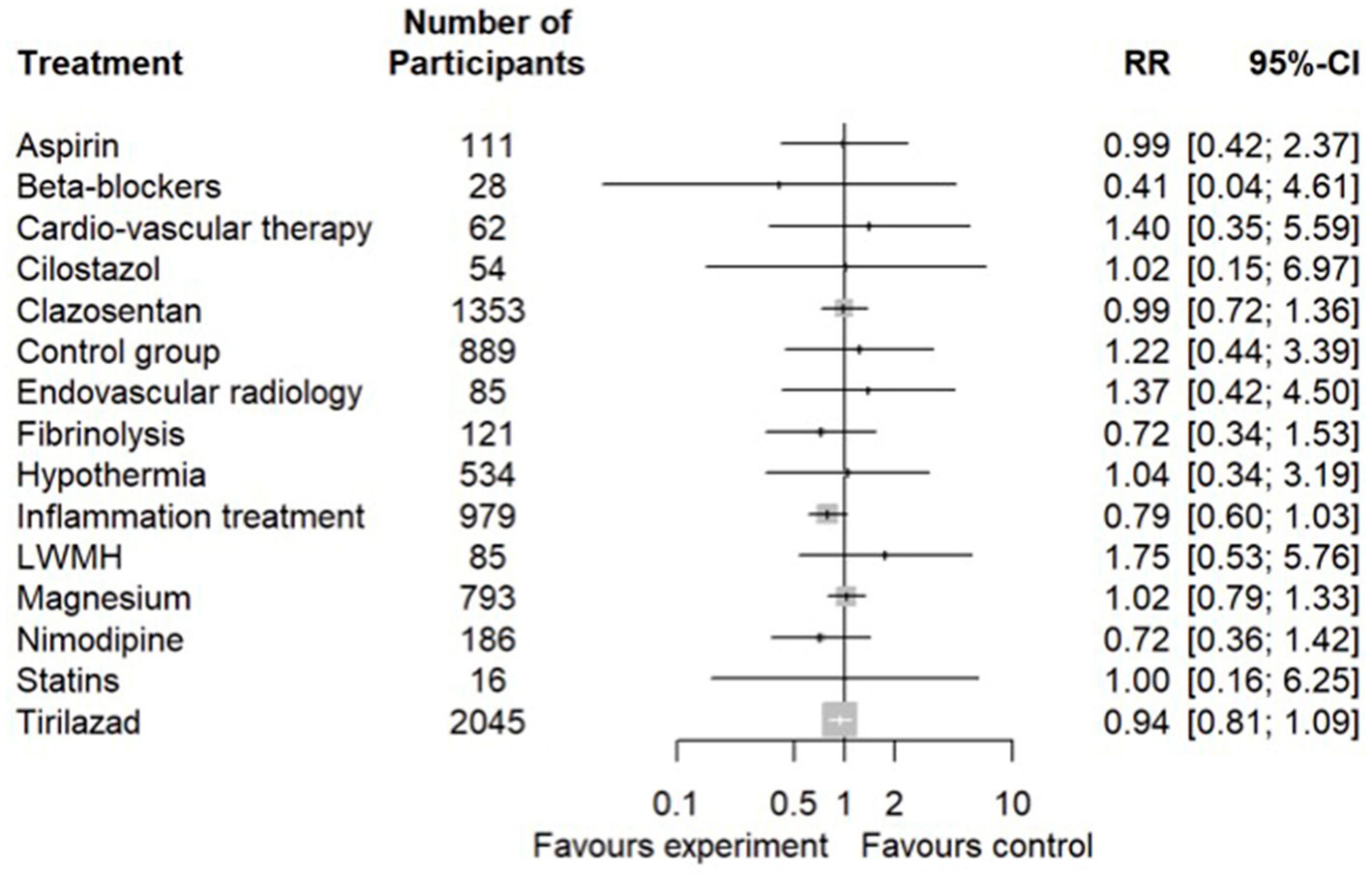
Primary analysis of mortality at 3 months. Network meta-analysis regarding preventive and curative therapies of vasospasm and brain ischemia in SAH patients on mortality. Primary analysis in randomized controlled trials. The number of patients involved in every strategy is displayed in the figure. LWMH, low-weight molecular heparin.

Thirty-five randomized controlled studies reporting mortality by 3 months were used in the network analysis with 16 different interventions (Supplementary Figure S4, Network studied for mortality): In the primary analysis, no strategy altered mortality (Figure 2). Strategy ranking is available in Table 1. There was a low heterogeneity between studies (*I*^2^ = 0, 95% CI [0–0.47]). In the secondary analysis (randomized controlled trials and cohorts), no strategy altered mortality (Supplementary Figure S5, Secondary analysis of mortality). No statistically significant publication bias was found regarding studies focusing on mortality (*p* = 0.052, Supplementary Figure S6, Funnel Plot Mortality).

**Table 1:** Ranking of interventions regarding mortality by 3 months.

	***P*-value**
Low-weight molecular heparin	0.77
Neuroradiology procedures	0.69
Cardiovascular therapies (hypertension and vascular expansion)	0.68
Standard of care	0.63
Magnesium	0.56
Placebo	0.55
Anti-endothelin	0.52
Cilostazol	0.51
Hypothermia	0.51
Aspirin	0.51
Statins	0.51
Tirilazad	0.45
Inflammation targeted therapies	0.29
Nimodipine	0.29
Fibrinolysis	0.28
Beta-blockers	0.24

### 3.4. Secondary outcomes

#### 3.4.1. Vasospasm and cerebral ischemia

**Figure 3:**
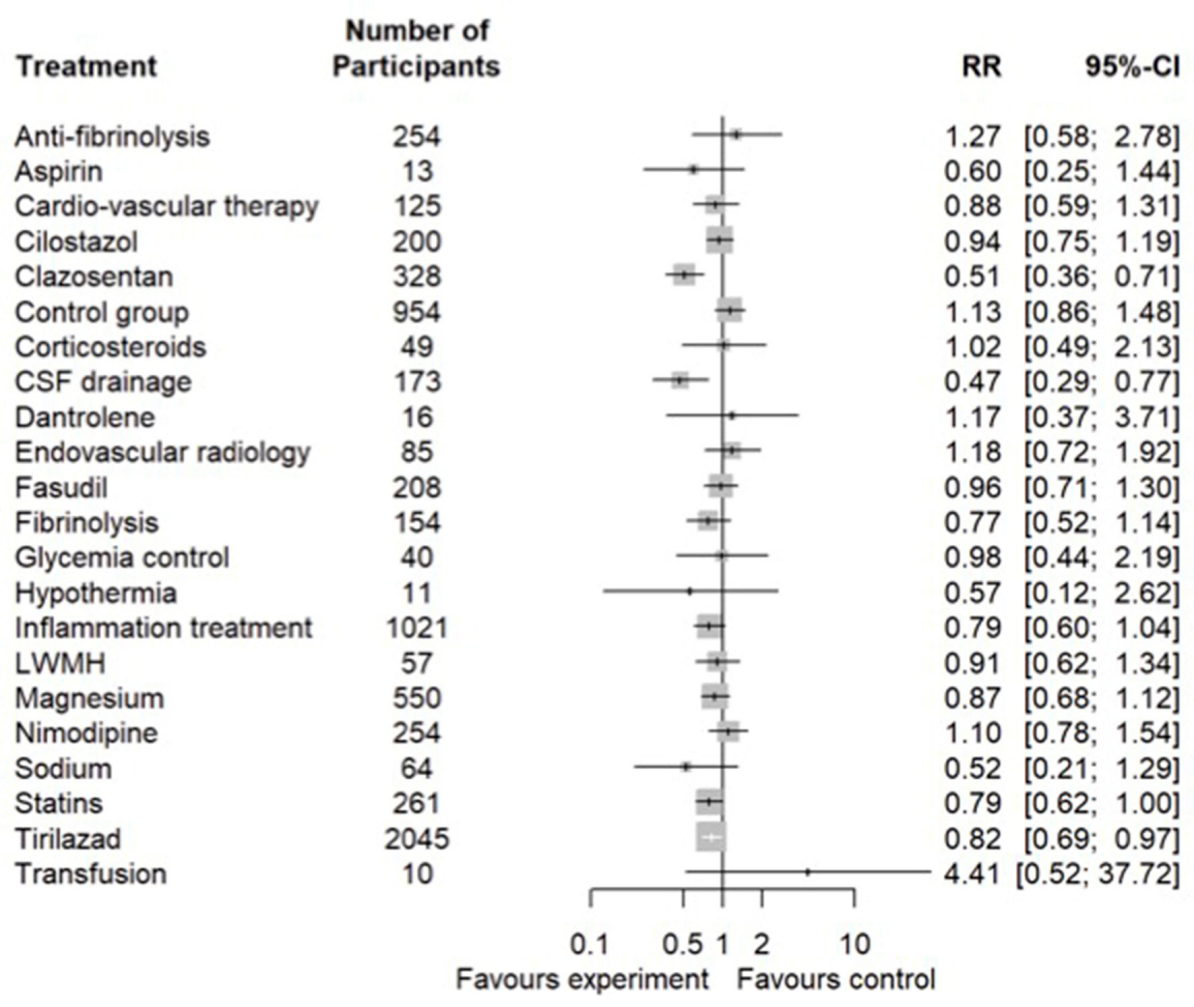
Vasospasm. Network meta-analysis regarding preventive and curative therapies of vasospasm in SAH. Primary analysis in randomized controlled trials. The number of patients involved in every strategy is displayed in the figure. LWMH, low-weight molecular heparin; CSF, cerebrospinal fluid.

A total of 6 interventional studies provided a comparison of 23 interventions (Supplementary Figure S7, Network for Vasospasm) with significant heterogeneity (*I*^2^ = 0.33,2 95% CI [0.01–0.54], Supplementary Figure S8, Funnel plot vasospasm). In the primary analysis, clazosentan (OR 0.51, 95% CI [0.36**–**0.71]), CSF drainage (OR 0.47, 95% CI [0.29–0.77]), statins (OR 0.79, 95% CI [0.62–1]), and tirilazad (OR 0.82, 95% CI [0.69–0.97]) significantly decreased the incidence of vasospasm (Figure 3). The ranking between strategies and interventions is available in Supplementary Table S9 (Ranking between strategies for vasospasm). In the secondary analysis, no interventions significantly decreased the incidence of vasospasm (Supplementary Figure S10, Secondary analysis vasospasm).

#### 3.4.2. Neurological outcome by 3 months

**Figure 4:**
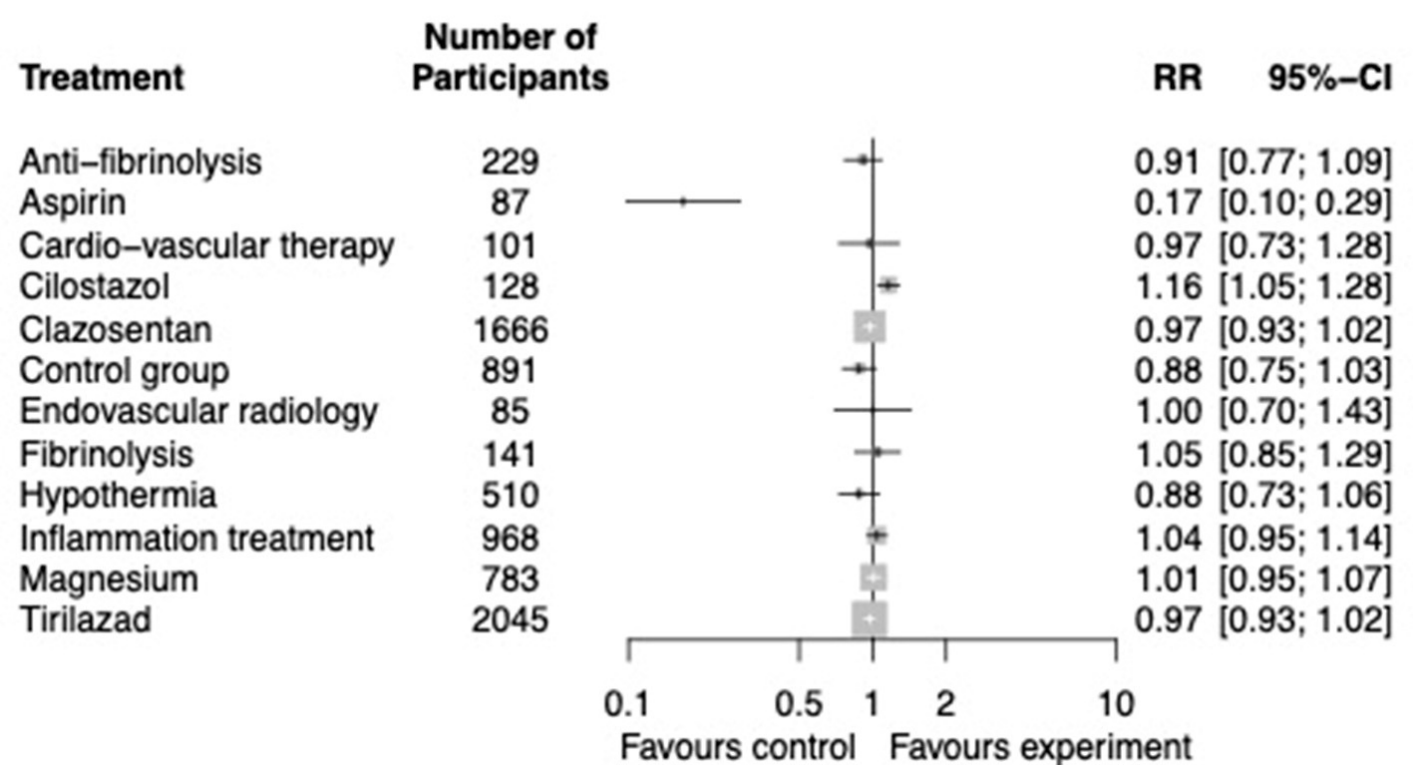
Neurological outcome by 3 months. Network meta-analysis regarding preventive and curative therapies on good or bad neurological recovery by 3 months. Primary analysis in randomized controlled trials. The number of patients involved in every strategy is displayed in the figure.

Thirty-two interventional studies enabled the comparison of 13 interventions (Supplementary Figure S11, Network for neurological outcome) without significant heterogeneity (*I*^2^ = 0, 95% CI [0–0.47]). In the primary analysis, only cilostazol was significantly associated with improved outcomes (OR 1.16, 95% CI [1.05–1.28]) (Figure 4). Ranking between interventions is available in Supplementary Table S12 (Ranking between interventions for neurological outcome). There was a significant publication bias regarding studies focusing on the neurological outcome by 3 months (*p* = 0.005, Supplementary Figure S13, Funnel plot neurological outcome). In the secondary analyses, cilostazol was still associated with improved outcomes (OR 2.97, 95% CI [1.39–6.32]), whereas aspirin (OR 0.03, 95% CI [0.01–0.08]), endovascular procedures (OR 0.46, 95% CI [0.26–0.8]), and hypothermia (OR 0.53, 95% CI [0.33–0.83]) were associated with worse outcomes (Supplementary Figure S14, Secondary analysis of neurological outcomes).

## 4. Discussion

### 4.1. Principal findings

In our network meta-analysis, among all curative or prophylactic strategies that have been tested in the last 30 years in subarachnoid hemorrhage patients, none appeared to decrease mortality by 3 months. Enteral cilostazol could improve neurological outcomes, but this should be confirmed in large randomized controlled studies. Statin, clazosentan, and cerebrospinal fluid drainage decreased the incidence of vasospasm.

Cilostazol is a selective inhibitor of phosphodiesterase 3 and has pleiotropic effects: anti-platelet, micro-vessel vasodilating, inhibition of reactive oxygen species production, inhibition of apoptosis, anti-inflammatory, anti-lipid peroxidation, and induction of NO synthesis (17). The underlying mechanisms of its potential effects are therefore difficult to assess and could be unrelated to vasospasm. Other meta-analyses confirmed the potential benefit of cilostazol in SAH (18, 19) and stroke patients (20). However, cilostazol is not available in all countries. Its benefit in stroke urgently deserves further investigation.

In our meta-analysis, we found many definitions of vasospasm or delayed cerebral ischemia: doppler with various thresholds, increased temperature, sodium variation, clinical impairment with the modified Glasgow Coma scale, CT scan, etc. The lack of standardization in the definition of vasospasm could explain the great variation in incidence in the studies. The primary analysis performed in randomized controlled trials enabled meta-analysis, and four different interventions significantly decreased the incidence of vasospasm. However, it is unclear why clazosentan and statins were not associated with improved outcomes, unlike cilostazol. One could argue that the adverse events of these treatments could counterbalance their potential benefit (13). Nonetheless, these conflicting results challenge the link between vasospasm and neurological recovery. Moreover, we believe that an international consensus regarding the definition of vasospasm and the selection of patients who could benefit from prophylactic/curative treatment is urgently needed.

Nimodipine is a cornerstone of treatment and is recommended in guidelines but was not a standard of care in every study that we analyzed (14). A meta-analysis performed in 2008 pointed out the benefit of calcium antagonists regarding neurological recovery (21). In another recent network meta-analysis (18), the authors reported that nimodipine decreased mortality. There are some differences between the present study and this meta-analysis (18). First, we decided to reject some studies performed before the 1990's. We assumed that practices could have drastically changed since then. Hence, the inclusion of studies in the 1980's (4) could clearly alter our results on nimodipine. It should be noted that several studies were performed in the 1980's where the authors used specific clinical scales to evaluate vasospasm-related neurologic features, which, to the best of our knowledge, have not reappeared in recent randomized controlled studies. In our meta-analysis, nimodipine did not appear to provide significant improvement. Moreover, its usefulness in the most severe patients requiring general anesthesia is questionable owing to its hypotensive effects (22) and its direct antagonism with norepinephrine which is often mandatory to preserve cerebral perfusion.

Cardiovascular strategies or, more recently, hypertensive therapy did not provide any benefit in our meta-analysis. To this date, induced hypertension has been scarcely evaluated in the literature. In the most recent randomized controlled study (23), the authors failed to include the expected number of patients probably because they considered it unethical not to increase arterial pressure in the setting of new neurological symptoms. Interestingly, in this study, patients could improve without induced hypertension and almost half of the patients in the elevated blood pressure group presented a serious adverse event (23). This strategy nonetheless warrants urgent appraisal in order to adequately assess its risk/benefit ratio. Finally, it is possible that the selection of patients who could benefit from point-of-care induced hypertension is difficult owing to the lack of consensus regarding the diagnosis and treatment of vasospasm.

### 4.2. Strengths and weaknesses of our study

The strengths of our study reside in its transparent design, the exhaustivity of strategies evaluated, a predefined research question, and the selection of both randomized controlled and observational studies. Our search string was not very sensitive because of the wide range of strategies tested in SAH patients and because vasospasm/cerebral ischemia is not specifically targeted by interventions (7). However, in addition to the literature search, we retrieved articles from reference lists, meta-analyses, and reverse snowballing. Because of the various settings in which all studies occurred, mortality by 3 months appeared as the most robust primary outcome. Finally, our meta-analysis describes many different interventions that are not discussed in the latest guidelines (14).

Our study also has weaknesses. First, we did not specifically look for unpublished data. Second, some strategies such as aspirin or cardiovascular management were poorly evaluated in randomized controlled studies. The strength of comparisons between studies is limited, but since very few strategies altered the outcome, this drawback has limited importance. We did not collect general demographic data, which could have enabled us to compare similarities between studies. Finally, our review stopped in November 2021. More recent studies could modify our findings.

### 4.3. Future research

After more than 30 years of clinical research, little achievement has been made in spite of the numerous attempts and various strategies tested to treat or prevent vasospasm and cerebral ischemia in SAH patients. A better understanding of the underlying neuro-inflammatory processes after the rupture of an intracranial aneurysm is urgently needed in order to propose new therapeutic strategies. Moreover, an adequate scientific evaluation of ongoing strategies such as induced hypertension therapy is also urgently needed owing to the lack of data in the literature. A consensus should be reached regarding the definition of vasospasm and delineate potential point-of-care strategies. Finally, cilostazol deserves urgent evaluation in order to confirm its efficacy in neurological recovery.

## Data availability statement

The original contributions presented in the study are included in the article/Supplementary material, further inquiries can be directed to the corresponding author.

## Author contributions

RC, BC, and LM designed the study, performed the literature search, collected data, and analyzed the results. RC, BC, YE, BL, and LM elaborated on data collection. BL performed the statistical analysis. RC and BL wrote the article. LM, BC, EG, YE, and AM critically amended the article. All authors contributed to the article and approved the submitted version.
