# Supplementary material for: A network meta-analysis of therapeutic and prophylactic management of vasospasm on aneurysmal subarachnoid hemorrhage outcomes
Source: Front Neurol. 2023 Aug 17;14:1217719. doi: 10.3389/fneur.2023.1217719 (PMC10469900; doi:10.3389/fneur.2023.1217719)
Supplement: Supplementary file 1 [file Data_Sheet_1.DOCX]

**S1. Text. Complete search algorithm**

('subarachnoid hemorrhage'/exp OR ('subarachnoid' AND 'hemorrhage') OR 'subarachnoid haemorrhage') AND [article]/lim AND ([english]/lim OR [french]/lim) AND [1980-2021]/py NOT ([cochrane review]/lim OR [systematic review]/lim OR [meta analysis]/lim)

AND ([embase]/lim OR [medline]/lim)

AND ('clinical article'/de OR 'clinical protocol'/de OR 'clinical trial'/de OR 'cohort analysis'/de OR 'comparative study'/de OR 'controlled clinical trial'/de OR 'controlled study'/de OR 'double blind procedure'/de OR 'feasibility study'/de OR 'intermethod comparison'/de OR 'major clinical study'/de OR 'multicenter study'/de OR 'observational study'/de OR 'outcomes research'/de OR 'phase 2 clinical trial'/de OR 'pilot study'/de OR 'prospective study'/de OR 'randomized controlled trial'/de OR 'retrospective study'/de)

NOT 'reversible cerebral vasoconstriction syndrome'/de NOT 'case report'/de AND [abstracts]/lim

AND ('clinical trial'/lnk OR 'complication'/lnk OR 'disease management'/lnk OR 'drug administration'/lnk OR 'drug combination'/lnk OR 'drug comparison'/lnk OR 'drug dose'/lnk OR 'drug therapy'/lnk OR 'intraarterial drug administration'/lnk OR 'intrathecal drug administration'/lnk OR 'intravenous drug administration'/lnk OR 'oral drug administration'/lnk OR 'subcutaneous drug administration'/lnk OR 'surgery'/lnk OR 'therapy'/lnk)

AND ('treatment outcome'/de OR 'outcome assessment'/de OR 'mortality'/de) AND ('female'/de OR 'male'/de) AND 'human'/de

| **S2. List of interventional studies included** | | | |  |  |  |
| --- | --- | --- | --- | --- | --- | --- |
| **Author** | **Title** | **Year** | **Neurological evolution by 3 months** | **Mortality by 3 months** | **Vasospasm** | **N. inclusion** |
| Juvela S. | Effect of nimodipine on platelet function in patients with subarachnoid hemorrhage | 1990 |  |  |  | 41 |
| Brandt L. | Does Nimodipine eliminate arterial hypertension as prognostic risk factor in subarachnoid haemorrhage? | 1991 |  |  |  | 274 |
| Ohman J. | Long-term effects of nimodipine on cerebral infarcts and outcome after aneurysmal sunarachnoid hemorrhage and surgery | 1991 |  |  |  | 213 |
| Tokiyoshi | Efficacy and Toxicity of Thromboxane Synthetase Inhibitor for Cerebral Vasospasm After Subarachnoid Hemorrhage | 1991 |  |  | x | 24 |
| Yanamoto | Therapeutic Trial of Cerebral Vasospasm with the Serine Protease Inhibitor, FUT-175, Administered in the Acute Stage after Subarachnoid Hemorrhage Experimental and Clinical Study | 1992 |  |  |  | 45 |
| Shibuya | Effect of AT877 on cerebralvasospasm afteraneurysmal subarachnoid hemorrhage | 1992 |  |  | x | 267 |
| Haley E.C. | A randomized controlled trial of high-dose intravenous nicardipine in aneurysmal subarachnoid hemorrhage | 1993 | x | x | x | 906 |
| Haman | Beta-Blockade in Acute Aneurysmal Subarachnoid Haemorrhage | 1993 |  |  |  | 25 |
| Mizoi | Prospective study on the prevention of cerebral vasospasm by intrathecal fibrinolytic therapy with tissue-type plasminogen activator | 1993 |  |  | x | 105 |
| Suzuki | Efficacy of Steroid Hormone in Solution for Intracranial Irrigation During Aneurysmal Surgery for Prevention of the Vasospasm Syndrome | 1994 |  |  | x | 123 |
| Shibuya M. | Effects of Prophylactic Intrathecal Administrations of Nicardipine on Vasospasm in Patients with Severe Aneurysmal Subarachnoïd Haemorrhage | 1994 |  |  | x | 141 |
| Seifert | Prevention of Delayed Ischaemic Deficits After Aneurysmal Subarachnoid Haemorrhage by Intrathecal Bolus Injection of Tissue Plasminogen Activator (rTPA) | 1994 |  | x | x | 120 |
| Usui | asospasm Prevention with Postoperative Intrathecal Thrombolytic Therapy: A Retrospective Comparison of Urokinase, Tissue Plasminogen Activator, and Cisternal Drainage Alone Clinical Study | 1994 |  | x |  | 111 |
| Haley | Phase II trial of tirilazad in aneurysmal subarachnoid hemorrhage | 1995 | x | x | x | 245 |
| Moriyama | bined Cisternal Drainage and Intrathecal Urokinase Injection Therapy for Prevention of Vasospasm in Patients with Aneurysmal Subarachnoid Hemorrhage | 1995 |  |  | x | 44 |
| Findlay | A randomized trial of intraoperative, intracisternal tissue plasminogen activator for the prevention of vasospasm. | 1995 | x | x | x | 100 |
| Kassell | Randomized, double-blind, vehicle-controlled trial of tirilazad mesylate in patients with aneurysmal subarachnoid hemorrhage: a cooperative study in Europe, Australia, and New Zealand | 1996 | x | x | x | 1015 |
| Asano | Effects of a hydroxyl radical scavenger on delayed ischemic neurological deficits following aneurysmal subarachnoid hemorrhage: results of a multicenter, placebo-controlled double-blind trial | 1996 | x | x |  | 162 |
| Haley | A randomized, double-blind, vehicle-controlled trial of tirilazad mesylate in patients with aneurysmal subarachnoid hemorrhage: a cooperative study in North America | 1997 | x | x | x | 897 |
| Kawai K. | Efficacy and risk of ventricular drainage in cases of grade V subarachnoid hemorrhage | 1997 |  |  |  | 93 |
| Mitosek-Sabbo | Peri-operative hemodilution in intracranial aneurysm surgery | 1998 |  |  |  | 80 |
| Vermeij | Impact of Medical Treatment on the Outcome of Patients After Aneurysmal Subarachnoid Hemorrhage | 1998 |  | x |  | 348 |
| Kaminogo | Combnation of Serine Protease Inhibitor FUT-175 and Thromboxane Synthetase Inhibitor OKY-046 Decreases Cerebral Vasospasm in Patients with Subarachnoid Hemorrhage | 1998 | x |  | x | 489 |
| Okamoto | Ameliorated Outcome of Subarachnoid Hemorrhage Patients Treated with Reduced Form Glutathione | 1998 | x | x | x | 147 |
| Saito | Neuroprotective Effect of an Antioxidant, Ebselen, in Patients with Delayed Neurological Deficits after Aneurysmal Subarachnoid Hemorrhage | 1998 |  |  |  | 286 |
| Elliott JP | Comparison of balloon angioplasty and papaverine infusion for the treatment of vasospasm following aneurysmal subarachnoid hemorrhage | 1998 |  |  | x | 52 |
| Polin RS | Intra-arterially Administered Papaverine for the Treatment of Symptomatic Cerebral Vasospasm | 1998 | x |  |  | 93 |
| Mori | Improved efficiency of hypervolemic therapy with inhibition of natriuresis by fludrocortisone in patients with aneurysmal subarachnoid hemorrhage | 1999 |  |  | x | 30 |
| Lanzino | Double-blind, randomized, vehicle-controlled study of high- dose tirilazad mesylate in women with aneurysmal subarachnoid hemorrhage. Part I. A cooperative study in Europe, Australia, New Zealand, and South Africa | 1999 | x | x | x | 819 |
| Lanzino | Double-blind, randomized, vehicle-controlled study of high-dose tirilazad mesylate in women with aneurysmal subarachnoid hemorrhage. Part II. A cooperative study in North America | 1999 | x | x | x | 823 |
| Karinen P. | Cost-effectiveness analysis of nimodipine treatment after aneurysmal subarachnoid hemorrhage and surgery | 1999 |  | x |  | 127 |
| Hindman | Mild Hypothermia as a Protective Therapy during Intracranial Aneurysm Surgery: A Randomized Prospective Pilot Trial | 1999 |  | x |  | 52 |
| Katoh H. | Clinical evaluation of the effect of percutaneous transluminal angioplasty and intra-arterial papaverine infusion for the treatment of vasospasm following aneurysmal subarachnoid hemorrhage | 1999 |  |  |  | 44 |
| Lennihan | Effect of Hypervolemic Therapy on Cerebral Blood Flow After Subarachnoid Hemorrhage A Randomized Controlled Trial | 2000 |  | x | x | 82 |
| Shaw M.D. | Efficacy and safety of the endothelinA/B receptor antagonist TAK-044 in treating subarachnoid hemorrhage: a report by the Steering Committee on behalf of the UK/Netherlands/Eire TAK-044 Subarachnoid Haemorrhage Study Group | 2000 | x | x |  | 420 |
| Roos | Antifibrinolytic treatment in subarachnoid hemorrhage A randomized placebo-controlled trial | 2000 | x |  |  | 462 |
| Hop | Randomized pilot trial of postoperative aspirin in subarachnoid hemorrhage | 2000 |  | x |  | 50 |
| Polin RS | Efficacy of transluminal angioplasty for the management of symptomatic cerebral vasospasm following aneurysmal subarachnoid hemorrhage | 2000 | x |  |  | 121 |
| Egge | Prophylactic Hyperdynamic Postoperative Fluid Therapy after Aneurysmal Subarachnoid Hemorrhage: A Clinical, Prospective, Randomized, Controlled Study | 2001 |  |  | x | 32 |
| Masaoka | Clinical Effect of Fasudil Hydrochloride for Cerebral Vasospasm Following Subarachnoid Hemorrhage | 2001 |  |  |  | 74 |
| Nakagomi | Cisternal Washing Therapy for the Prevention of Cerebral Vasospasm Following Aneurysmal Subarachnoid Hemorrhage | 2001 |  | x | x | 182 |
| Veyna | Magnesium sulfate therapy after aneurysmal subarachnoid hemorrhage | 2002 | x |  | x | 36 |
| Chia | Magnesium: a useful adjunct in the prevention of cerebral vasospasm following aneurysmal subarachnoid haemorrhage | 2002 |  | x | x | 23 |
| Hillman | Immediate administration of tranexamic acid and reduced incidence of early rebleeding after aneurysmal subarachnoid hemorrhage: a prospective randomized study | 2002 |  |  | x | 505 |
| Moro | Prophylactic Management of Excessive Natriuresis With Hydrocortisone for Efficient Hypervolemic Therapy After Subarachnoid Hemorrhage | 2003 |  |  | x | 28 |
| Hamada | Effect on Cerebral Vasospasm of Coil Embolization Followed by Microcatheter Intrathecal Urokinase Infusion Into the Cisterna Magna A Prospective Randomized Study | 2003 |  |  | x | 110 |
| Siironen | No effect of enoxaparin on outcome of aneurysmal subarachnoid hemorrhage: a randomized, double-blind, placebo-controlled clinical trial | 2003 |  | x |  | 170 |
| Badjatia N. | Preliminary experience with intra-arterial nicardipine as a treatment for cerebral vasospasm | 2004 |  |  |  | 24 |
| Yoneda | Does eicosapentaenoic acid (EPA) inhibit cerebral vasospasm in patients after aneurysmal subarachnoid hemorrhage? | 2004 |  |  | x | 101 |
| Suarez | Effect of human albumin administration on clinical outcome and hospital cost in patients with subarachnoid hemorrhage | 2004 | x |  | x | 84 |
| Wurm | Reduction of ischemic sequelae following spontaneous subarachnoid hemorrhage: a double-blind, randomized comparison of enoxaparin versus placebo | 2004 |  |  | x | 117 |
| Kawamoto | Effectiveness of the head-shaking method combined with cisternal irrigation with urokinase in preventing cerebral vasospasm after subarachnoid hemorrhage | 2004 |  |  | x | 225 |
| Findlay | Cohort study of intraventricular thrombolysis with recombinant tissue plasminogen activator for aneurysmal intraventricular hemorrhage | 2004 |  |  |  | 30 |
| Andaluz N. | Fenestration of the lamina terminalis as a valuable adjunct in aneurysm surgery | 2004 |  |  | x | 106 |
| Klimo P. | Marked reduction of cerebral vasospasm with lumbar drainage of cerebrospinal fluid after subarachnoid hemorrhage | 2004 |  |  | x | 167 |
| Smith MJ | Blood transfusion and increased risk for vasospasm and poor outcome after subarachnoid hemorrhage | 2004 |  |  | x | 494 |
| Van den Bergh | Magnesium Sulfate in Aneurysmal Subarachnoid Hemorrhage. A Randomized Controlled Trial | 2005 | x |  |  | 249 |
| Boet | Intravenous magnesium sulfate to improve outcome after aneurysmal subarachnoid hemorrhage: interim report from a pilot study | 2005 | x | x |  | 45 |
| Kim | Reduction of Pulmonary Edema After SAH With a Pulmonary Artery Catheter-Guided Hemodynamic Management Protocol | 2005 |  |  | x | 453 |
| Kasuya H. | APPLICATION OF NICARDIPINE PROLONGED-RELEASE IMPLANTS: ANALYSIS OF 97 CONSECUTIVE PATIENTS WITH ACUTE SUBARACHNOID HEMORRHAGE | 2005 | x | x | x | 97 |
| Todd | Mild Intraoperative Hypothermia during Surgery for Intracranial Aneurysm | 2005 | x | x |  | 1000 |
| Vajkoczy P. | Clazosentan (AXV-034343), a selective endothelin A receptor antagonist, in the prevention of cerebral vasospasm following severe aneurysmal subarachnoid hemorrhage: results of a randomized, double-blind, placebo-controlled, multicenter Phase IIa study | 2005 |  |  | x | 32 |
| D’Ambrosio A. L. | Decompressive hemicraniectomy for poor-grade aneurysmal subarachnoid hemorrhage patients with associated intracerebral hemorrhage: clinical outcome and quality of life assessment | 2005 | x | x |  | 22 |
| Lynch JR | Simvastatin Reduces Vasospasm After Aneurysmal Subarachnoid Hemorrhage | 2005 |  |  | x | 39 |
| Parra A. | EFFECT OF PRIOR STATIN USE ON FUNCTIONAL OUTCOME AND DELAYED VASOSPASM AFTER ACUTE ANEURYSMAL SUBARACHNOID HEMORRHAGE: A MATCHED CONTROLLED COHORT STUDY | 2005 |  |  | x | 60 |
| Tseng MY | Effects of Acute Treatment With Pravastatin on Cerebral Vasospasm, Autoregulation, and Delayed Ischemic Deficits After Aneurysmal Subarachnoid Hemorrhage A Phase II Randomized Placebo-Controlled Trial | 2005 |  |  | x | 80 |
| Schmid-Elsaesser | INTRAVENOUS MAGNESIUM VERSUS NIMODIPINE IN THE TREATMENT OF PATIENTS WITH ANEURYSMAL SUBARACHNOID HEMORRHAGE: A RANDOMIZED STUDY | 2006 |  |  | x | 104 |
| Prevedello | Magnesium sulfate: role as possible attenuating factor in vasospasm morbidity | 2006 |  |  | x | 72 |
| Stippler | Magnesium infusion for vasospasm prophylaxis after subarachnoid hemorrhage | 2006 |  | x | x | 76 |
| Wong | Intravenous Magnesium Sulfate After Aneurysmal Subarachnoid Hemorrhage: A Prospective Randomized Pilot Study | 2006 |  |  | x | 60 |
| Van den Berghe | Randomized Controlled Trial of Acetylsalicylic Acid in Aneurysmal Subarachnoid Hemorrhage | 2006 | x | x |  | 161 |
| Zhao | Effect of Fasudil Hydrochloride, a Protein Kinase Inhibitor, on Cerebral Vasospasm and Delayed Cerebral Ischemic Symptoms After Aneurysmal Subarachnoid Hemorrhage —Results of a Randomized Trial of Fasudil Hydrochloride Versus Nimodipine— | 2006 |  |  | x | 72 |
| Akyuz M. | The effects of fenestration of the interpeduncular cistern membrane arousted to the opening of lamina terminalis in patients with ruptured ACoA aneurysms: a prospective, comparative study | 2006 |  |  |  | 145 |
| Barth M. | Effect of nicardipine prolonged-release implants on cerebral vasospasm and clinical outcome after severe aneurysmal subarachnoid hemorrhage: A prospective, randomized, double-blind phase IIa study | 2007 |  |  | x | 29 |
| Katayama | A Randomized Controlled Trial of Hydrocortisone Against Hyponatremia in Patients With Aneurysmal Subarachnoid Hemorrhage | 2007 |  |  | x | 71 |
| Mura J. | Improved outcome in high-grade aneurysmal subarachnoid hemorrhage by enhancement of endogenous clearance of cisternal blood clots: a prospective study that demonstrates the role of lamina terminalis fenestration combined with modern microsurgical cisternal blood evacuation | 2007 |  |  | x | 123 |
| Bilotta F. | The Effect of Intensive Insulin Therapy on Infection Rate, Vasospasm, Neurologic Outcome, and Mortality in Neurointensive Care Unit After Intracranial Aneurysm Clipping in Patients With Acute Subarachnoid Hemorrhage A Randomized Prospective Pilot Trial | 2007 |  |  | x | 78 |
| Muroi | Magnesium sulfate in the management of patients with aneurysmal subarachnoid hemorrhage: a randomized, placebo-controlled, dose-adapted trial | 2008 |  | x | x | 58 |
| Berger | Influence of early antioxidant supplements on clinical evolution and organ function in critically ill cardiac surgery, major trauma, and subarachnoid hemorrhage patients | 2008 |  | x |  | 21 |
| Muroi | Combined Therapeutic Hypothermia and Barbiturate Coma Reduces Interleukin-6 in the Cerebrospinal Fluid After Aneurysmal Subarachnoid Hemorrhage | 2008 |  |  |  | 15 |
| McGregor | Effect of Nitrous Oxide on Neurologic and Neuropsychological Function after Intracranial Aneurysm Surgery | 2008 | x | x |  | 1046 |
| Macdonald R.L. | Clazosentan to Overcome Neurological Ischemia and Infarction Occurring After Subarachnoid Hemorrhage (CONSCIOUS-1) | 2008 | x |  | x | 409 |
| Starke | Impact of a Protocol for Acute Antifibrinolytic Therapy on Aneurysm Rebleeding After Subarachnoid Hemorrhage | 2008 | x |  | x | 248 |
| Yamada | Effectiveness of combining continuous cerebrospinal drainage and intermittent intrathecal urokinase injection therapy in preventing symptomatic vasospasm following aneurysmal subarachnoid haemorrhage | 2008 | x | x | x | 69 |
| Hänggi D. | The effect of lumboventricular lavage and simultaneous low-frequency head-motion therapy after severe subarachnoid hemorrhage: results of a single center prospective Phase II trial | 2008 |  | x | x | 40 |
| Kwon O. Y. | The utility and benefits of external lumbar CSF drainage after endovascular coiling on aneurysmal subarachnoid hemorrhage | 2008 |  |  | x | 107 |
| CHOU SHY | A Randomized, Double-Blind, Placebo-Controlled Pilot Study of Simvastatin in Aneurysmal Subarachnoid Hemorrhage | 2008 |  |  | x | 39 |
| Kern M. | Statins may not protect against vasospasm in subarachnoid haemorrhage | 2008 |  |  | x | 130 |
| Kerz t. | A case control study of statin and magnesium administration in patients after aneurysmal subarachnoid hemorrhage: incidence of delayed cerebral ischemia and mortality | 2008 |  |  | x | 100 |
| Kramer AH | STATIN USE WAS NOT ASSOCIATED WITH LESS VASOSPASM OR IMPROVED OUTCOME AFTER SUBARACHNOID HEMORRHAGE | 2008 |  |  | x | 150 |
| Latorre JGS | Effective Glycemic Control With Aggressive Hyperglycemia Management Is Associated With Improved Outcome in Aneurysmal Subarachnoid Hemorrhage | 2008 |  |  | x | 332 |
| Zwienengerg-Lee M. | Effect of Prophylactic Transluminal Balloon Angioplasty on Cerebral Vasospasm and Outcome in Patients With Fisher Grade III Subarachnoid Hemorrhage Results of a Phase II Multicenter, Randomized, Clinical Trial | 2008 | x | x | x | 170 |
| Shah | Super-Selective Intra-arterial Magnesium Sulfate in Combination With Nicardipine for the Treatment of Cerebral Vasospasm in Patients With Subarachnoid Hemorrhage | 2009 |  |  | x | 43 |
| Akdemir | Magnesium Sulfate Therapy for Cerebral Vasospasm After Aneurysmal Subarachnoid Hemorrhage | 2009 |  | x | x | 83 |
| Friedlich | Retrospective analysis of parenteral magnesium sulfate administration in decreased incidence of clinical and neuroradiological cerebral vasospasm: a single center experience | 2009 |  |  | x | 85 |
| Mutoh | Performance of Bedside Transpulmonary Thermodilution Monitoring for Goal-Directed Hemodynamic Management After Subarachnoid Hemorrhage | 2009 | x | x |  | 100 |
| Yoshimoto | Cilostazol May Prevent Cerebral Vasospasm Following Subarachnoid Hemorrhage | 2009 |  |  | x | 50 |
| Hänggi | A Multimodal Concept in Patients after Severe Aneurysmal Subarachnoid Hemorrhage: Results of a Controlled Single Centre Prospective Randomized Multimodal Phase I/II Trial on Cerebral Vasospasm | 2009 | x |  | x | 20 |
| Munakata | EFFECT OF A FREE RADICAL SCAVENGER, EDARAVONE, IN THE TREATMENT OF PATIENTS WITH ANEURYSMAL SUBARACHNOID HEMORRHAGE | 2009 | x | x | x | 91 |
| McGirt MJ | Simvastatin for the prevention of symptomatic cerebral vasospasm following aneurysmal subarachnoid hemorrhage: a single-institution prospective cohort study | 2009 |  |  | x | 340 |
| Moskowitz SI | Pre-hemorrhage statin use and the risk of vasospasm following aneurysmal subarachnoid hemorrhage | 2009 |  |  | x | 308 |
| Vergouwen MDI | Biologic effects of simvastatin in patients with aneurysmal subarachnoid hemorrhage: a double-blind, placebo-controlled randomized trial | 2009 |  | x | x | 32 |
| Thiele RH | Strict Glucose Control Does Not Affect Mortality after Aneurysmal Subarachnoid Hemorrhage | 2009 |  |  | x | 834 |
| Westermaier | Prophylactic intravenous magnesium sulfate for treatment of aneurysmal subarachnoid hemorrhage: A randomized, placebo-controlled, clinical study | 2010 |  |  | x | 107 |
| Wong | Intravenous Magnesium Sulphate for Aneurysmal Subarachnoid Hemorrhage (IMASH) A Randomized, Double-Blinded, Placebo-Controlled, Multicenter Phase III Trial | 2010 |  |  | x | 327 |
| Satoh | Prevention of Symptomatic Vasospasm by Continuous Cisternal Irrigation with Mock-CSF Containing Ascorbic Acid and Mg2+ | 2010 |  |  | x | 103 |
| Gomis | Randomized, double-blind, placebo-controlled, pilot trial of high-dose methylprednisolone in aneurysmal subarachnoid hemorrhage | 2010 |  |  | x | 95 |
| Anei | Effectiveness of Brain Hypothermia Treatment in Patients With Severe Subarachnoid Hemorrhage — Comparisons at a Single Facility | 2010 | x | x |  | 35 |
| Yamamoto | Efficacy of low-dose tissue- plasminogen activator intracisternal administration for the prevention of cerebral vasospasm after subarachnoid hemorrhage | 2010 | x | x |  | 60 |
| Kawaguchi | Effects of a Short-acting b1 Receptor Antagonist Landiolol on Hemodynamics and Tissue Injury Markers in Patients With Subarachnoid Hemorrhage Undergoing Intracranial Aneurysm Surgery | 2010 |  | x |  | 56 |
| Barth M. | Feasibility of intraventricular nicardipine prolonged release implants in patients following aneurysmal subarachnoid haemorrhage. | 2011 |  |  | x | 47 |
| Hauer | Early continuous hypertonic saline infusion in patients with severe cerebrovascular disease | 2011 |  |  |  | 38 |
| Lu N. | Intraventricular Nicardipine for Aneurysmal Subarachnoid Hemorrhage Related Vasospasm: Assessment of 90 Days Outcome | 2011 | x | x |  | 27 |
| Suzuki | Cilostazol Improves Outcome after Subarachnoid Hemorrhage: A Preliminary Report | 2011 |  |  | x | 100 |
| Schneider U.C. | The use of nicardipine prolonged release implants (NPRI) in microsurgical clipping after aneurysmal subarachnoid haemorrhage: comparison with endovascular treatment | 2011 |  |  | x | 81 |
| Macdonald R.L. | Clazosentan, an endothelin receptor antagonist, in patients with aneurysmal subarachnoid haemorrhage undergoing surgical clipping: a randomised, double-blind, placebo-controlled phase 3 trial (CONSCIOUS-2) | 2011 | x | x |  | 1147 |
| Khatri R. | Impact of Percutaneous Transluminal Angioplasty for Treatment of Cerebral Vasospasm on Subarachnoid Hemorrhage Patient Outcomes | 2011 |  |  | x | 146 |
| Dorhout Mees | Magnesium for aneurysmal subarachnoid haemorrhage (MASH-2): a randomised placebo-controlled trial | 2012 | x | x |  | 1203 |
| Jeon | Intravenous Magnesium Infusion for the Prevention of Symptomatic Cerebral Vasospasm after Aneurysmal Subarachnoid Hemorrhage | 2012 |  |  | x | 82 |
| Macdonald R.L. | Randomized Trial of Clazosentan in Patients With Aneurysmal Subarachnoid Hemorrhage Undergoing Endovascular Coiling | 2012 | x | x |  | 571 |
| Masa | Beneficial Effect of Selective Intra-arterial Infusion of Fasudil Hydrochloride as a Treatment of Symptomatic Vasospasm Following SAH | 2012 |  |  |  | 31 |
| Al-Tamini Y. Z. | Lumbar drainage of cerebrospinal fluid after aneurysmal subarachnoid hemorrhage | 2012 |  |  |  | 210 |
| Sanchez-Pena P. | Atorvastatin decreases computed tomography and S100-assessed brain ischemia after subarachnoid aneurysmal hemorrhage: A comparative study | 2012 |  |  | x | 278 |
| Aburto-Murrieta Y. | Endovascular Treatment: Balloon Angioplasty Versus Nimodipine Intra-arterial for Medically Refractory Cerebral Vasospasm Following Aneurysmal Subarachnoid Hemorrhage | 2012 | x | x | x | 30 |
| Kerz T | Eff ect of intraarterial papaverine or nimodipine on vessel diameter in patients with cerebral vasospasm after subarachnoid hemorrhage | 2012 |  |  |  | 30 |
| Bradford | A randomised controlled trial of induced hypermagnesaemia following aneurysmal subarachnoid haemorrhage | 2013 |  | x | x | 162 |
| Ibrahim | The Effects of Fluid Balance and Colloid Administration on Outcomes in Patients with Aneurysmal Subarachnoid Hemorrhage: A Propensity Score-Matched Analysis | 2013 |  |  |  | 123 |
| Kuwabara | Association of early post-procedure hemodynamic management with the outcomes of subarachnoid hemorrhage patients | 2013 |  |  |  | 6418 |
| Nakagawa | Early Inhibition of Natriuresis Suppresses Symptomatic Cerebral Vasospasm in Patients with Aneurysmal Subarachnoid Hemorrhage | 2013 |  |  | x | 103 |
| Senbokuya | Effects of cilostazol on cerebral vasospasm after aneurysmal subarachnoid hemorrhage: a multicenter prospective, randomized, open-label blinded end point trial | 2013 | x | x | x | 109 |
| Etminan | Prospective, Randomized, Open-Label Phase II Trial on Concomitant Intraventricular Fibrinolysis and Low- Frequency Rotation After Severe Subarachnoid Hemorrhage | 2013 | x | x | x | 60 |
| Litrico | Intraventricular fibrinolysis for severe aneurysmal intraventricular hemorrhage: a randomized controlled trial and meta-analysis | 2013 | x |  | x | 19 |
| Brinjikji | Rescue Treatment of Thromboembolic Complications During Endovascular Treatment of Cerebral Aneurysms | 2013 |  |  |  | 310 |
| de Aguiar P. H. P. | Removal of clots in subarachnoid space could reduce the vasospasm after subarachnoid hemorrhage | 2013 |  |  | x | 30 |
| Uozumi Y. | Decompressive craniectomy in patients with aneurysmal subarachnoid hemorrhage: a single-center matched-pair analysis | 2013 |  |  |  | 112 |
| Garg K. | Role of simvastatin in prevention of vasospasm and improving functional outcome after aneurysmal sub-arachnoid hemorrhage: a prospective, randomized, double-blind, placebocontrolled pilot trial | 2013 |  |  | x | 38 |
| Festic E | Blood Transfusion is an Important Predictor of Hospital Mortality Among Patients with Aneurysmal Subarachnoid Hemorrhage | 2013 |  |  | x | 316 |
| Mutoh | Early Intensive Versus Minimally Invasive Approach to Postoperative Hemodynamic Management After Subarachnoid Hemorrhage | 2014 | x |  |  | 160 |
| Tagami | Effect of Triple-H Prophylaxis on Global End-Diastolic Volume and Clinical Outcomes in Patients with Aneurysmal Subarachnoid Hemorrhage | 2014 |  |  |  | 178 |
| Singh | The effect of intravenous interleukin-1 receptor antagonist on inflammatory mediators in cerebrospinal fluid after subarachnoid haemorrhage: a phase II randomised controlled trial | 2014 |  |  |  | 13 |
| Kimura | Cilostazol Administration with Combination Enteral and Parenteral Nutrition Therapy Remarkably Improves Outcome After Subarachnoid Hemorrhage | 2014 |  |  | x | 130 |
| Karnatovskaia | Effect of Prolonged Therapeutic Hypothermia on Intracranial Pressure, Organ Function, and Hospital Outcomes Among Patients with Aneurysmal Subarachnoid Hemorrhage | 2014 |  |  |  | 35 |
| Muehlschlegel | Dantrolene for cerebral vasospasm after subarachnoid haemorrhage: a randomised double blind placebo-controlled safety trial | 2014 |  |  | x | 31 |
| Kramer | Intraventricular Tissue Plasminogen Activator in Subarachnoid Hemorrhage Patients: A Prospective, Randomized, Placebo- Controlled Pilot Trial | 2014 |  |  |  | 12 |
| Kirkpatrick PJ | Simvastatin in aneurysmal subarachnoid haemorrhage (STASH): a multicentre randomised phase 3 trial | 2014 |  |  |  | 803 |
| Kumar MA | Red Blood Cell Transfusion Increases the Risk of Thrombotic Events in Patients with Subarachnoid Hemorrhage | 2014 |  |  | x | 205 |
| Chalouhi N. | Endovascular management of cerebral vasospasm following aneurysm rupture: Outcomes and predictors in 116 patients | 2014 |  |  |  | 116 |
| Avdagic | Impact of Comorbidity on Early Outcome of Patients with Subarachnoid Hemorrhage Caused by Cerebral Aneurysm Rupture | 2015 |  |  |  | 50 |
| Togashi | Randomized Pilot Trial of Intensive Management of Blood Pressure or Volume Expansion in Subarachnoid Hemorrhage (IMPROVES).1 | 2015 |  |  | x | 20 |
| Ghodsi | Comparative Efficacy of Meloxicam and Placebo in Vasospasm of Patients with Subarachnoid Hemorrhage | 2015 |  |  | x | 81 |
| Kurumatsu | Is Hypothermia Helpful in Severe Subarachnoid Hemorrhage? An Exploratory Study on Macro Vascular Spasm, Delayed Cerebral Infarction and Functional Outcome after Prolonged Hypothermia | 2015 |  |  | x | 36 |
| Cengiz S. L. | The role of fenestration of the lamina terminalis on symptomatic vasospasm after aneurysmal subarachnoid hemorrhage: a clinical research | 2015 | x |  |  | 72 |
| Park S. | The effectiveness of lumbar cerebrospinal fluid drainage to reduce the cerebral vasospasm after surgical clipping for aneurysmal subarachnoid hemorrhage | 2015 |  |  | x | 234 |
| Zhao B. | Primary decompressive craniectomy for poor-grade middle cerebral artery aneurysms with associated intracerebral hemorrhage | 2015 |  |  | x | 38 |
| Zheng S. -F. | Keyhole approach combined with external ventricular drainage for ruptured, poor-grade, anterior circulation cerebral aneurysms | 2015 | x | x | x | 103 |
| Togashi | Randomized Pilot Trial of Intensive Management of Blood Pressure or Volume Expansion in Subarachnoid Hemorrhage (IMPROVES). 2 | 2015 |  |  | x | 20 |
| Luo R | Combination Treatment of Atorvastatin and Fasudil Ameliorate Cerebral Vasospasm after Subarachnoid Hemorrhage | 2015 |  |  | x | 121 |
| Nassiri | A Propensity Score-Matched Study of the Use of Non-steroidal Anti-inflammatory Agents Following Aneurysmal Subarachnoid Hemorrhage | 2016 |  |  | x | 178 |
| Matsuda | Effect of Cilostazol on Cerebral Vasospasm and Outcome in Patients with Aneurysmal Subarachnoid Hemorrhage: A Randomized, Double-Blind, Placebo-Controlled Trial | 2016 | x |  | x | 148 |
| Ehlert | Molsidomine for the prevention of vasospasm-related delayed ischemic neurological deficits and delayed brain infarction and the improvement of clinical outcome after subarachnoid hemorrhage: a single-center clinical observational study | 2016 |  | x |  | 74 |
| Chang | Beta Blockade and Clinical Outcome in Aneurysmal Subarachnoid Haemorrhage | 2016 |  |  | x | 200 |
| Panczykowski | Prophylactic Antiepileptics and Seizure Incidence Following Subarachnoid Hemorrhage A Propensity Score–Matched Analysis | 2016 |  |  |  | 353 |
| Diringer MN | Effect of high-dose simvastatin on cerebral blood flow and static autoregulation in subarachnoid hemorrhage | 2016 |  |  | x | 25 |
| Drevet | Impact of restrictive fluid protocol on hypoxemia after aneurysmal subarachnoid hemorrhage | 2017 | x |  | x | 120 |
| Czorlich | Impact of dexamethasone in patients with aneurysmal subarachnoid haemorrhage | 2017 |  |  |  | 306 |
| Mohney | A propensity score analysis of the impact of Dexamethasone use on delayed cerebral ischemia and poor functional outcomes following subarachnoid hemorrhage | 2017 |  |  | x | 309 |
| Choi | Feasibility and Safety of Mild Therapeutic Hypothermia in Poor- Grade Subarachnoid Hemorrhage: Prospective Pilot Study | 2017 | x | x | x | 22 |
| Human | A Randomized Trial of Brief Versus Extended Seizure Prophylaxis After Aneurysmal Subarachnoid Hemorrhage | 2017 |  |  |  | 84 |
| Roelz | Stereotactic Catheter Ventriculocisternostomy for Clearance of Subarachnoid Hemorrhage A Matched Cohort Study | 2017 | x |  |  | 80 |
| Naraoka M. | Long-acting statin for aneurysmal subarachnoid hemorrhage: A randomized, double-blind, placebo-controlled trial | 2017 |  |  | x | 108 |
| Kumar MA | The Effects of Red Blood Cell Transfusion on Functional Outcome after Aneurysmal Subarachnoid Hemorrhage | 2017 |  |  | x | 421 |
| Leal-Noval SR | Red blood cell transfusion guided by near infrared spectroscopy in neurocritically ill patients with moderate or severe anemia | 2017 |  |  | x | 23 |
| Bercker | Hydroxyethyl starch for volume expansion after subarachnoid haemorrhage and renal function: Results of a retrospective analysis | 2018 |  |  | x | 276 |
| Gathier | Induced Hypertension for Delayed Cerebral Ischemia After Aneurysmal Subarachnoid Hemorrhage A Randomized Clinical Trial | 2018 | x | x |  | 41 |
| Galea | Reduction of inflammation after administration of interleukin-1 receptor antagonist following aneurysmal subarachnoid hemorrhage: results of the Subcutaneous Interleukin-1Ra in SAH (SCIL-SAH) study | 2018 |  |  | x | 136 |
| Zhang C. | Magnesium sulfate in combination with nimodipine for the treatment of subarachnoid hemorrhage: a randomized controlled clinical study | 2018 |  |  | x | 120 |
| Rahmanian | Fresh Frozen Plasma versus Albumin in Treatment of Cerebral Vasospasm in Subarachnoid Hemorrhage: A Historical Cohort Study | 2018 |  |  |  | 364 |
| Sugimoto | Cilostazol decreases duration of spreading depolarization and spreading ischemia after aneurysmal subarachnoid hemorrhage | 2018 |  |  | x | 48 |
| Woo | Randomized, placebo-controlled, double- blind, pilot trial to investigate safety and efficacy of Cerebrolysin in patients with aneurysmal subarachnoid hemorrhage | 2019 |  |  | x | 50 |
| Post | Short-term tranexamic acid treatment reduces in-hospital mortality in aneurysmal sub-arachnoid hemorrhage: A multicenter comparison study | 2019 |  |  |  | 509 |
| Labeyrie | Distal Balloon Angioplasty of Cerebral Vasospasm Decreases the Risk of Delayed Cerebral Infarction | 2019 |  |  |  | 392 |
| Chen | Use of single versus multiple vasodilator agents in the treatment of cerebral vasospasm: is more better than less? | 2020 |  | x |  | 116 |
| Fang | The effectiveness of lumbar cerebrospinal fluid drainage in aneurysmal subarachnoid hemorrhage with different bleeding amounts | 2020 | x |  | x | 193 |
| Gàl | Assessment of two prophylactic fluid strategies in aneurysmal subarachnoid hemorrhage: A randomized trial | 2020 |  |  | x | 96 |
| Post | Ultra-early tranexamic acid after subarachnoid haemorrhage (ULTRA): a randomised controlled trial | 2021 |  |  |  | 955 |
| Katagai | Effect of Surgical Arachnoid Plasty on Functional Outcome in Aneurysmal Subarachnoid Hemorrhage | 2021 | x |  | x | 178 |
| Yoshikane | Aggressive Intraoperative Cisternal Clot Removal After Clipping Aneurismal Subarachnoid Hemorrhage in Elderly Patients | 2021 | x |  | x | 40 |
| Takeuchi | Intravenous Hydrogen Therapy With Intracisternal Magnesium Sulfate Infusion in Severe Aneurysmal Subarachnoid Hemorrhage | 2021 | x | x | x | 25 |

**S3. Figure. Number of studies included per decade**

**S4. Figure. Network of interventions regarding mortality**

**S5. Figure. Secondary analysis (randomized controlled trials and cohorts) on mortality**

**Legend:** LWMH: Low Weight Molecular Heparin. CSF: Cerebro-Spinal Fluid. NO: Nitrogen Monoxide.

**S6. Figure. Funnel Plot regarding the studies on mortality**

**S7. Figure. Network of interventions regarding vasospasm**

**S8. Funnel Plot regarding the studies on vasospasm**

**S9. Table. Ranking of interventions regarding vasospasm**

|  | **P-score** |
| --- | --- |
| Cerebrospinal liquid drainage | 0.94 |
| Anti-endothelin | 0.88 |
| Natremia management | 0.81 |
| Aspirin | 0.80 |
| Hypothermia | 0.73 |
| Inflammation targeted therapies | 0.67 |
| Fibrinolysis | 0.67 |
| Statins | 0.62 |
| Tirilazad | 0.59 |
| Cardiovascular therapies (hypertension, vascular expansion) | 0.57 |
| Magnesium | 0.55 |
| Glycemia management | 0.47 |
| Low Weight Molecular Heparin | 0.46 |
| Corticosteroids | 0.36 |
| Dantrolene | 0.32 |
| Placebo | 0.32 |
| Cilostazol | 0.32 |
| Fasudil | 0.30 |
| Anti-fibrinolytics | 0.27 |
| Standard of care | 0.27 |
| Neuroradiology procedures | 0.25 |
| Nimodipine | 0.24 |
| Transfusion | 0.08 |

**S10. Figure. Secondary analysis on vasospasm**

**Legend:** LWMH: Low Weight Molecular Heparin. CSF: Cerebro-Spinal Fluid.

**S11. Figure. Network of interventions on neurological outcomes**

**S12. Table. Ranking of interventions regarding neurological outcome by 3 months**

|  | ***p*-value** |
| --- | --- |
| Cilostazol | 0.95 |
| Inflammation targeted therapies - endothelin | 0.75 |
| Fibrinolysis | 0.70 |
| Magnesium | 0.65 |
| Placebo | 0.62 |
| Neuroradiology procedures | 0.58 |
| Cardio-vascular therapies (hypertension, vascular expansion) | 0.51 |
| Anti-endothelin | 0.47 |
| Tirilazad | 0.46 |
| Anti-fibrinolytics | 0.33 |
| Hypothermia | 0.25 |
| Standard of care | 0.23 |
| Aspirin | 0.00 |

**S13. Funnel plot of studies on neurological outcomes**

**S14. Secondary analysis on neurological outcomes**

**Legend:** LWMH: Low Weight Molecular Heparin. NO: Nitrogen Monoxide.
